# Hardy type inequalities on the sphere

**DOI:** 10.1186/s13660-017-1424-x

**Published:** 2017-06-24

**Authors:** Xiaomei Sun, Fan Pan

**Affiliations:** College of Science, Huazhong Agricultural University, Wuhan, 430070 China

**Keywords:** Hardy type inequality, sphere, best constant

## Abstract

In this paper, we consider the $L^{p}$-Hardy inequalities on the sphere. By the divergence theorem, we establish the $L^{p}$-Hardy inequalities on the sphere. Furthermore, we also obtain their best constants. Our results can be regarded as the extension of Xiao’s (J. Math. Inequal. 10:793-805, 2016).

## Introduction

The classical Hardy inequality states that for $N\geq 3$ and $p>1$

1 $$ \int_{R^{N}}\vert \nabla u\vert ^{p}\,dx\geq \biggl\vert \frac{N-p}{p}\biggr\vert ^{p} \int_{R^{N}}\frac{\vert u\vert ^{p}}{\vert x\vert ^{p}}\,dx, $$

with $u\in C_{0}^{\infty }(R^{N})\setminus \{0\}$ and $\vert \frac{N-p}{p}\vert ^{p}$ the best constant. In recent years, many papers have been dedicated to improved versions of the above inequality because of its application to singular problems. We see [2–6] and the references therein. Hardy inequalities are a subfamily of the Caffarelli-Kohn-Nirenberg inequalities. In a Riemannian manifold, the knowledge of the validity of these inequalities and their best constants allows us to obtain qualitative properties on the manifold [7–10].

Recently, Carron [11] studied the weighted $L^{2}$-Hardy inequalities on a Riemannian manifold under some geometric assumptions on the weighted function *ρ* and obtained the following inequality:

$$\int_{M}\rho^{\alpha }\vert \nabla u\vert ^{2}\,dx\geq \biggl\vert \frac{C+\alpha -1}{2}\biggr\vert ^{2} \int_{M}\rho^{\alpha }\frac{\vert u\vert ^{2}}{\rho^{2}}\,dx, $$

where the weighted function *ρ* satisfies $\vert \nabla \rho \vert =1$ and $\Delta \rho \geq \frac{C}{\rho }$. In [12], Grillo obtained Hardy, Rellich and Sobolev inequalities in homogeneous spaces. Recently, Kombe and Özaydin [13] extended Carron’s results to the general case $p\neq 2$. Moreover, they obtained the sharp versions of improved Hardy inequalities and an improved Rellich inequality in hyperbolic spaces. By the divergence theorem and careful choices of a vector field, D’Ambrosio and Dipierro [14] proved a sufficient criterion to obtain $L^{p}$-Hardy inequalities on Riemannian manifolds. That is, if *ρ* satisfies $-\Delta_{p} \rho \geq 0$, then the following Hardy inequality was obtained:

$$C \int_{M}\vert \nabla \rho \vert ^{p} \frac{\vert u\vert ^{p}}{\rho^{p}}\,dV_{g}\leq \int _{M}\vert \nabla u\vert ^{p}\,dV_{g},\quad \forall u\in C_{0}^{\infty }(M). $$

Very recently, by a similar approach that appeared in [13], Xiao [1] studied the $L^{2}$-Hardy inequality and the Rellich inequality on the sphere, and obtained their best constants. Yang, Su and Kong [15] considered the $L^{p}$-Hardy inequalities on a complete, simply connected Riemannian manifold with negative curvature. They obtained the sharp constants of Hardy and Rellich inequalities related to the geodesic distance. In this paper, we aim to extend Xiao’s results [1] to a general case.

## Our main results

Our main result is the following $L^{p}$-Hardy inequality on the sphere.

### Theorem 1

*Let* $N\geq 3$, $1< p< N$, $q\in S^{N}$, *then there exists a positive constant* $C>0$ *such that for all* $f\in C^{\infty } ( S^{N} ) $, *we have for* $2\leq p< N$,

2 $$\begin{aligned}& C \int_{S^{N}}\frac{\vert f\vert ^{p}}{\sin ^{p-2}d(x,q)}\,dV+ \int_{S^{N}}\vert \nabla_{S}f\vert ^{p}\,dV \\& \quad \geq \biggl( \frac{N-p}{p} \biggr) ^{p} \int_{S^{N}} \biggl( \frac{\vert f\vert ^{p}}{d ^{p}(x,q)}+\frac{\vert f\vert ^{p}}{ ( \pi -d(x,q)) ^{p}} \biggr)\,dV \end{aligned}$$

*for* $1< p<2$,

3 $$\begin{aligned}& C \int_{S^{N}}\vert f\vert ^{p}\,dV+ \int_{S^{N}}\vert \nabla_{S}f\vert ^{p}\,dV \\& \quad \geq \biggl( \frac{N-p}{p} \biggr) ^{p} \int_{S^{N}} \biggl( \frac{\vert f\vert ^{p}}{d^{p}(x,q)}+\frac{\vert f\vert ^{p}}{ ( \pi -d(x,q)) ^{p}} \biggr)\,dV, \end{aligned}$$

*where* $d(x,q)$ *is the geodesic distance of* *x* *and* *q*. *Moreover*, $( \frac{N-p}{p} ) ^{p}$ *is the best constant*.

### Remark 2

When $p=2$, inequality (3) was obtained by Xiao [1]. When $1< p<2$, $\int_{S^{N}}\frac{\vert f\vert ^{p}}{\sin ^{p-2}d(x,q)}\,dV$ cannot control the right-hand side of (3) since $\sin ^{p-2}d(x,q)$ is large enough when *x* is close to *q*. Therefore, we use $\int_{S^{N}}\vert f\vert ^{p}\,dV$ as the left-hand side of the inequality instead of $\int_{S^{N}}\frac{\vert f\vert ^{p}}{\sin ^{p-2}d(x,q)}\,dV$.

Although our approach is similar to Xiao’s [1], the appearance of general *p* makes the calculation more complicated, especially for the existence of the constant *C* in Theorem 1.

## Preliminaries and notations

Let $S^{N}=\{x=(x_{1},x_{2},\ldots,x_{n+1})\in R^{N+1};\vert x\vert =1\}$ be the unit sphere of dimension *N*. Let $(\theta_{1},\theta_{2}, \ldots, \theta_{N})$ be the angular variables on $S^{N}$. For simplicity, we define $\theta_{N}=\theta $, where $x_{N+1}=\vert x\vert \cos \theta_{N}$. By polar coordinates associated with *θ*, we get

$$\int_{S^{N}}f\,dV= \int_{S^{N-1}} \int_{0}^{\pi }f\sin ^{N-1}\theta \,d\sigma\, d \theta, $$

where *dσ* is the canonical measure of the unit sphere $S^{N-1}$. We say that a function *f* on $S^{N}$ is an angular function if *f* depends only on *θ*. In this case,

$$\Delta_{S}f=\sin ^{1-N}\theta \frac{d}{d\theta } \biggl( \sin ^{N-1}\theta \frac{df}{d \theta } \biggr) $$

and

$$\bigl\vert \nabla_{S}f(\theta)\bigr\vert =\bigl\vert f^{\prime}(\theta)\bigr\vert . $$

See [1]. For more basic properties on the sphere, we refer to [16].

## The proof of Theorem 1

Now we give the proof of Theorem 1.

Let $f=\rho^{\gamma }\varphi $, $\rho =\sin \theta $, $\gamma =- \frac{N-p}{p}$, by the calculation that appeared in [13], one has

$$\begin{aligned} \vert \nabla_{S}f\vert ^{p} =&\bigl\vert \nabla_{S}\bigl(\rho^{\gamma }\bigr)\varphi +\rho^{\gamma} \nabla_{S}\varphi \bigr\vert ^{p} \\ \geq& \bigl\vert \nabla_{S}\bigl(\rho^{\gamma }\bigr)\varphi \bigr\vert ^{p}+p\bigl\vert \nabla_{S}\bigl( \rho^{\gamma }\bigr)\varphi \bigr\vert ^{p-2}\vert \nabla_{S}\bigl(\rho^{\gamma }\bigr)\varphi \cdot \rho^{\gamma }\nabla_{S}\varphi, \end{aligned}$$

then integration by parts gives

$$\begin{aligned} \int_{S^{N}}\vert \nabla_{S}f\vert ^{p}\,dV \geq \vert \gamma \vert ^{p} \int_{S^{N}} \rho^{\gamma p-p}\vert \nabla_{S}\rho \vert ^{p}\vert \varphi \vert ^{p}\,dV-\frac{\vert \gamma \vert ^{p-2}\gamma }{\gamma p-p+2} \int_{S^{N}}\Delta_{S} \bigl( \rho^{\gamma p-p+2} \bigr) \vert \varphi \vert ^{p}\,dV. \end{aligned}$$

Since

$$\vert \nabla_{S}\rho \vert =\vert \nabla_{S}\sin \theta \vert =\vert \cos \theta \vert $$

and

$$\Delta_{S} \bigl( \rho^{\gamma p-p+2} \bigr) =\Delta_{S} \bigl( \sin ^{2-N} \theta \bigr) =(N-2)\sin ^{2-N}\theta, $$

one has

$$\begin{aligned} \int_{S^{N}}\vert \nabla_{S}f\vert ^{p}\,dV \geq \biggl( \frac{N-p}{p} \biggr) ^{p} \int_{S^{N}}\frac{\vert \cos \theta \vert ^{p}\vert \varphi \vert ^{p}}{\sin ^{N}\theta }\,dV- \biggl( \frac{N-p}{p} \biggr) ^{p-1} \int_{S^{N}}\frac{\vert \varphi \vert ^{p}}{\sin ^{N-2}\theta }\,dV. \end{aligned}$$

While

$$\vert \cos \theta \vert ^{p}=\bigl\vert \cos ^{2}\theta \bigr\vert ^{\frac{p}{2}}= \bigl( 1-\sin ^{2}\theta \bigr) ^{\frac{p}{2}}\geq 1-\frac{p}{2}\sin ^{2}\theta\quad \mbox{for } p \geq 2 $$

and

$$\vert \cos \theta \vert ^{p}\geq \cos ^{2}\theta \quad \mbox{for } 1< p\leq 2. $$

The previous inequality can be written as follows:

$$\begin{aligned}& \int_{S^{N}}\vert \nabla_{S}f\vert ^{p}\,dV \\& \quad \geq \biggl( \frac{N-p}{p} \biggr) ^{p} \int_{S^{N}}\frac{\vert \varphi \vert ^{p}}{\sin ^{N}\theta }\,dV \\& \qquad {}- \biggl( \biggl( \frac{N-p}{p} \biggr) ^{p-1} +\min \biggl\{ \frac{p}{2},1 \biggr\} \biggl( \frac{N-p}{p} \biggr) ^{p} \biggr) \int_{S^{N}}\frac{\vert \varphi \vert ^{p}}{\sin ^{N-2}\theta }\,dV. \end{aligned}$$

Define $C(N,p)= ( \frac{N-p}{p} ) ^{p-1} +\min \{ \frac{p}{2},1 \} ( \frac{N-p}{p} ) ^{p}$, then

4 $$\begin{aligned}& C(N,p) \int_{S^{N}}\frac{\vert f\vert ^{p}}{\sin ^{p-2}\theta }\,dV+ \int_{S^{N}}\vert \nabla_{S}f\vert ^{p}\,dV \\& \quad \geq \biggl( \frac{N-p}{p} \biggr) ^{p} \int_{S^{N}}\frac{\vert f\vert ^{p}}{\sin ^{p}\theta }\,dV. \end{aligned}$$

In order to get our result, we rewrite the above inequality as

5 $$\begin{aligned}& C(N,p) \int_{S^{N}}\frac{\vert f\vert ^{p}}{\sin ^{p-2}\theta }\,dV+ \int_{S^{N}}\vert \nabla_{S}f\vert ^{p}\,dV \\& \quad \geq \biggl( \frac{N-p}{p} \biggr) ^{p} \int_{S^{N}} \biggl( \frac{\vert f\vert ^{p}}{ \theta^{p}}+\frac{\vert f\vert ^{p}}{(\pi -\theta)^{p}} \biggr)\,dV \\& \qquad {}+ \biggl( \frac{N-p}{p} \biggr) ^{p} \int_{S^{N}}\frac{\vert f\vert ^{p}}{\sin ^{p-2} \theta } \biggl( \frac{1}{\sin ^{2}\theta }- \frac{\sin ^{p-2}\theta }{\theta ^{p}}-\frac{\sin ^{p-2}\theta }{(\pi -\theta)^{p}} \biggr)\,dV. \end{aligned}$$

As we know, for $p>2$,

$$\begin{aligned} \lim_{\theta \rightarrow 0^{+}} \biggl( \frac{1}{\sin ^{2}\theta }-\frac{\sin ^{p-2}\theta }{\theta^{p}}- \frac{\sin ^{p-2}\theta }{(\pi -\theta)^{p}} \biggr) =&\lim_{\theta \rightarrow 0^{+}}\frac{\theta^{p}-\sin ^{p}\theta }{ \theta^{p+2}} \\ =&\lim _{\theta \rightarrow 0^{+}}\frac{1- ( \frac{\sin \theta }{\theta } ) ^{p}}{\theta^{2}} \\ \geq& \lim_{\theta \rightarrow 0^{+}}\frac{1- ( \frac{\sin \theta }{\theta } ) ^{2}}{\theta^{2}} \\ =&\frac{1}{3}>0, \end{aligned}$$

and for $p=2$,

$$\lim_{\theta \rightarrow 0^{+}} \biggl( \frac{1}{\sin ^{2}\theta }-\frac{\sin ^{p-2}\theta }{\theta^{p}}- \frac{\sin ^{p-2}\theta }{(\pi -\theta)^{p}} \biggr) = \frac{1}{3}-\frac{1}{ \pi }>0. $$

Also, by a similar calculation, one has for $p>2$

$$\lim_{\theta \rightarrow \pi^{-}} \biggl( \frac{1}{\sin ^{2}\theta }-\frac{\sin ^{p-2}\theta }{\theta^{p}}- \frac{\sin ^{p-2}\theta }{(\pi - \theta)^{p}} \biggr) \geq \frac{1}{3}>0, $$

and for $p=2$

$$\lim_{\theta \rightarrow \pi^{-}} \biggl( \frac{1}{\sin ^{2}\theta }-\frac{\sin ^{p-2}\theta }{\theta^{p}}- \frac{\sin ^{p-2}\theta }{(\pi - \theta)^{p}} \biggr) \geq \frac{1}{3}-\frac{1}{\pi }>0. $$

Therefore, there exist a constant $\theta_{1}>0$ small enough and $\theta_{2}<\pi $ close to *π* such that for any $\theta \in (0, \theta_{1}]\cup [\theta_{2},\pi)$, one has that

$$\frac{1}{\sin ^{2}\theta }-\frac{\sin ^{p-2}\theta }{\theta^{p}}-\frac{\sin ^{p-2}\theta }{(\pi -\theta)^{p}}>0 \quad \mbox{for } p\geq 2. $$

Thus from inequality (5), one has

$$\begin{aligned}& C(N,p) \int_{S^{N}}\frac{\vert f\vert ^{p}}{\sin ^{p-2}\theta }\,dV+ \int_{S^{N}}\vert \nabla_{S}f\vert ^{p}\,dV \\& \quad \geq \biggl( \frac{N-p}{p} \biggr) ^{p} \int_{S^{N}} \biggl( \frac{\vert f\vert ^{p}}{ \theta^{p}}+\frac{\vert f\vert ^{p}}{(\pi -\theta)^{p}} \biggr)\,dV \\& \qquad {}+ \biggl( \frac{N-p}{p} \biggr) ^{p} \int_{S^{N}\cap ( [0,\theta_{1}]\cup [\theta_{2},\pi ] ) }\frac{\vert f\vert ^{p}}{\sin ^{p-2}\theta } \biggl( \frac{1}{\sin ^{2}\theta }- \frac{\sin ^{p-2}\theta }{ \theta^{p}}-\frac{\sin ^{p-2}\theta }{(\pi -\theta)^{p}} \biggr)\,dV \\& \qquad {}+ \biggl( \frac{N-p}{p} \biggr) ^{p} \int_{S^{N}\cap [\theta_{1},\theta_{2}]}\frac{\vert f\vert ^{p}}{\sin ^{p-2} \theta } \biggl( \frac{1}{\sin ^{2}\theta }- \frac{\sin ^{p-2}\theta }{\theta ^{p}}-\frac{\sin ^{p-2}\theta }{(\pi -\theta)^{p}} \biggr)\,dV \\& \quad \geq \biggl( \frac{N-p}{p} \biggr) ^{p} \int_{S^{N}} \biggl( \frac{\vert f\vert ^{p}}{ \theta^{p}}+\frac{\vert f\vert ^{p}}{(\pi -\theta)^{p}} \biggr)\,dV-C_{1} \int_{S ^{N}}\frac{\vert f\vert ^{p}}{\sin ^{p-2}\theta }\,dV, \end{aligned}$$

where $C_{1}=\sup_{\theta \in [\theta_{1},\theta_{2}]} ( \frac{1}{\sin ^{2}\theta }-\frac{\sin ^{p-2}\theta }{\theta^{p}}-\frac{\sin ^{p-2} \theta }{(\pi -\theta)^{p}} ) $.

Let $C=C(N,p)+C_{1}$, one has that for $p\geq 2 $

$$\begin{aligned} C \int_{S^{N}}\frac{\vert f\vert ^{p}}{\sin ^{p-2}\theta }\,dV+ \int_{S^{N}}\vert \nabla_{S}f\vert ^{p}\,dV \geq \biggl( \frac{N-p}{p} \biggr) ^{p} \int_{S^{N}} \biggl( \frac{\vert f\vert ^{p}}{ \theta^{p}}+\frac{\vert f\vert ^{p}}{(\pi -\theta)^{p}} \biggr)\,dV , \end{aligned}$$

which is exactly inequality (2).

While for $1< p<2$, we get from inequality (4) that

6 $$\begin{aligned}& C(N,p) \int_{S^{N}}\frac{\vert f\vert ^{p}}{\sin ^{p-2}\theta }\,dV+ \int_{S^{N}}\vert \nabla_{S}f\vert ^{p}\,dV \\& \quad \geq \biggl( \frac{N-p}{p} \biggr) ^{p} \int_{S^{N}} \biggl( \frac{\vert f\vert ^{p}}{ \theta^{p}}+\frac{\vert f\vert ^{p}}{(\pi -\theta)^{p}} \biggr)\,dV \\& \qquad {}+ \biggl( \frac{N-p}{p} \biggr) ^{p} \int_{S^{N}}\vert f\vert ^{p} \biggl( \frac{1}{\sin ^{p}\theta }-\frac{1}{\theta^{p}}-\frac{1}{(\pi -\theta)^{p}} \biggr)\,dV. \end{aligned}$$

By a similar calculation, we get that for $1< p<2$

$$\begin{aligned} -\frac{1}{(\pi -\theta)^{p}} \leq& \biggl( \frac{1}{\sin ^{p}\theta }-\frac{1}{ \theta^{p}}- \frac{1}{(\pi -\theta)^{p}} \biggr) \\ =&\frac{1- ( \frac{\sin \theta }{\theta } ) ^{p}}{\sin ^{p}\theta }- \frac{1}{(\pi -\theta)^{p}}\leq \frac{1- ( \frac{\sin \theta }{ \theta } ) ^{2}}{\sin ^{2}\theta } -\frac{1}{(\pi -\theta)^{p}}. \end{aligned}$$

Since

$$\lim_{\theta \rightarrow 0^{+}}-\frac{1}{(\pi -\theta)^{p}}=-\frac{1}{ \pi^{p}},\qquad \lim _{\theta \rightarrow 0^{+}}\frac{1- ( \frac{\sin \theta }{\theta } ) ^{2}}{\sin ^{2}\theta } -\frac{1}{(\pi -\theta)^{p}}=\frac{1}{3}- \frac{1}{\pi^{p}}, $$

we get that there exists a constant $\theta_{1}>0$ small enough such that

$$\sup_{\theta \in [0,\theta_{1}]}\biggl\vert \biggl( \frac{1}{\sin ^{p} \theta }- \frac{1}{\theta^{p}}-\frac{1}{(\pi -\theta)^{p}} \biggr) \biggr\vert < + \infty. $$

Furthermore, by a similar calculation, we get that there exists a constant $\theta_{2}$ close to *π* such that

$$\sup_{\theta \in [\theta_{2},\pi ]}\biggl\vert \biggl( \frac{1}{\sin ^{p}\theta }- \frac{1}{\theta^{p}}-\frac{1}{(\pi -\theta)^{p}} \biggr) \biggr\vert < + \infty. $$

Therefore, we get that

$$C_{2}:=\sup_{\theta \in [0,\pi ]}\biggl\vert \biggl( \frac{1}{\sin ^{p} \theta }-\frac{1}{\theta^{p}}-\frac{1}{(\pi -\theta)^{p}} \biggr) \biggr\vert < + \infty. $$

Then, by inequality (6), one has

$$\begin{aligned}& C(N,p) \int_{S^{N}}\frac{\vert f\vert ^{p}}{\sin ^{p-2}\theta }\,dV+ \int_{S^{N}}\vert \nabla_{S}f\vert ^{p}\,dV \\& \quad \geq \biggl( \frac{N-p}{p} \biggr) ^{p} \int_{S^{N}} \biggl( \frac{\vert f\vert ^{p}}{ \theta^{p}}+\frac{\vert f\vert ^{p}}{(\pi -\theta)^{p}} \biggr)\,dV -C_{2} \biggl( \frac{N-p}{p} \biggr) ^{p} \int_{S^{N}}\vert f\vert ^{p}\,dV. \end{aligned}$$

Let $C=C(N,p)+C_{2} ( \frac{N-p}{p} ) ^{p}$, we get for $1< p<2$

$$\begin{aligned} C \int_{S^{N}}\vert f\vert ^{p}\,dV+ \int_{S^{N}}\vert \nabla_{S}f\vert ^{p}\,dV \geq \biggl( \frac{N-p}{p} \biggr) ^{p} \int_{S^{N}} \biggl( \frac{\vert f\vert ^{p}}{\theta^{p}}+\frac{\vert f\vert ^{p}}{( \pi -\theta)^{p}} \biggr)\,dV, \end{aligned}$$

which is exactly inequality (3).

Now we prove that $( \frac{N-p}{p} ) ^{p}$ is the best constant of inequalities (2) and (3).

Let $\varphi (t)\in [0,1]$ be a cut-off function such that $\varphi (t) \equiv 1$ for $\vert t\vert \leq 1$; $\varphi (t)\equiv 0$ for $\vert t\vert >2$. Define $H(t)=1-\varphi (t)$ and

$$f_{\epsilon }(\theta)=H \biggl( \frac{\theta }{\epsilon } \biggr) \theta^{\frac{p-N}{p}}\quad \mbox{for } 0< \theta \leq \pi;\qquad f_{ \epsilon }(\theta)=0 \quad \mbox{for } \theta =0. $$

Then we have

7 $$\begin{aligned}& \int_{S^{N}}\frac{\vert f_{\epsilon }\vert ^{p}}{\sin ^{p-2}\theta }\,dV=\bigl\vert S^{N-1}\bigr\vert \int_{\epsilon }^{\pi }H^{p} \biggl( \frac{\theta }{\epsilon } \biggr) \theta^{p-N}\sin ^{N-p+1}\theta \,d\theta \leq \bigl\vert S^{N-1}\bigr\vert \frac{\pi^{2}- \epsilon^{2}}{2}, \end{aligned}$$

8 $$\begin{aligned}& \int_{S^{N}}\vert f_{\epsilon }\vert ^{p}\,dV=\bigl\vert S^{N-1}\bigr\vert \int_{\epsilon }^{\pi }H ^{p} \biggl( \frac{\theta }{\epsilon } \biggr) \theta^{p-N}\sin ^{N-1}\theta \,d\theta \leq \bigl\vert S^{N-1}\bigr\vert \frac{\pi^{p}-\epsilon^{p}}{p}, \end{aligned}$$

9 $$\begin{aligned}& \int_{S^{N}}\frac{\vert f_{\epsilon }\vert ^{p}}{\theta^{p}}\,dV=\bigl\vert S^{N-1}\bigr\vert \int_{\epsilon }^{\pi }H^{p} \biggl( \frac{\theta }{\epsilon } \biggr) \theta^{-N}\sin ^{N-1}\theta \,d\theta \geq \bigl\vert S^{N-1}\bigr\vert \int_{2\epsilon } ^{\pi }\theta^{-N}\sin ^{N-1} \theta \,d\theta, \end{aligned}$$

10 $$\begin{aligned}& \int_{S^{N}}\vert \nabla_{S}f_{\epsilon }\vert ^{p}\,dV \\& \quad =\bigl\vert S^{N-1}\bigr\vert \int_{\epsilon }^{\pi }\biggl\vert \frac{1}{\epsilon }H^{\prime} \biggl( \frac{\theta }{\epsilon } \biggr) \theta^{\frac{p-N}{p}} + \frac{p-N}{q}H \biggl( \frac{\theta }{\epsilon } \biggr) \theta^{- \frac{N}{p}}\biggr\vert ^{p}\sin ^{N-1}\theta \,d\theta \\& \quad =\bigl\vert S^{N-1}\bigr\vert \int_{\epsilon }^{2\epsilon }\biggl\vert \frac{1}{\epsilon }H ^{\prime} \biggl( \frac{\theta }{\epsilon } \biggr) \theta^{\frac{p-N}{p}} + \frac{p-N}{p}H \biggl( \frac{\theta }{\epsilon } \biggr) \theta^{- \frac{N}{p}}\biggr\vert ^{p}\sin ^{N-1}\theta \,d\theta \\& \qquad {}+\bigl\vert S^{N-1}\bigr\vert \biggl( \frac{N-p}{p} \biggr) ^{p} \int_{2\epsilon }^{\pi } \biggl\vert H \biggl( \frac{\theta }{\epsilon } \biggr) \theta^{-\frac{N}{p}}\biggr\vert ^{p}\sin ^{N-1} \theta \,d\theta \\& \quad \leq C_{1}\max_{t\in [0,2]}\bigl\vert H^{\prime}(t)\bigr\vert +C_{2} \int_{\epsilon } ^{2\epsilon }\theta^{-1}\,d\theta +\bigl\vert S^{N-1}\bigr\vert \biggl( \frac{N-p}{p} \biggr) ^{p} \int_{2\epsilon }^{\pi }\theta^{-N}\sin ^{N-1} \theta \,d\theta \\& \quad \leq C_{4}+\bigl\vert S^{N-1}\bigr\vert \biggl( \frac{N-p}{p} \biggr) ^{p} \int_{2\epsilon } ^{\pi }\theta^{-N}\sin ^{N-1} \theta \,d\theta. \end{aligned}$$

Therefore, for $p\geq 2$, from inequalities (7), (9) and (10), one has

$$\begin{aligned}& \inf_{f\in C^{\infty }(S^{N})}\frac{C\int_{S^{N}}\frac{\vert f\vert ^{p}}{\sin ^{p-2}\theta }\,dV+\int_{S^{N}}\vert \nabla_{S}f\vert ^{p}\,dV}{\int_{S^{N}}\frac{\vert f\vert ^{p}}{ \theta^{p}}\,dV} \\& \quad \leq \frac{C\int_{S^{N}}\frac{\vert f_{\epsilon }\vert ^{p}}{\sin ^{p-2}\theta }\,dV+\int_{S^{N}}\vert \nabla_{S_{\epsilon }}f\vert ^{p}\,dV}{\int_{S^{N}} \frac{\vert f_{\epsilon }\vert ^{p}}{\theta^{p}}\,dV} \\& \quad \leq \frac{\vert S^{N-1}\vert \frac{\pi^{2}-\epsilon^{2}}{2}+ C_{4}+\vert S^{N-1}\vert ( \frac{N-p}{p} ) ^{p}\int_{2\epsilon }^{\pi }\theta^{-N}\sin ^{N-1}\theta \,d\theta }{\vert S^{N-1}\vert \int_{2\epsilon }^{\pi }\theta^{-N}\sin ^{N-1}\theta \,d\theta }, \end{aligned}$$

then passing to the limit as $\epsilon \rightarrow 0^{+}$, we have

11 $$ \inf_{f\in C^{\infty }(S^{N})}\frac{C\int_{S^{N}}\frac{\vert f\vert ^{p}}{\sin ^{p-2}\theta }\,dV+\int_{S^{N}}\vert \nabla_{S}f\vert ^{p}\,dV}{\int_{S^{N}}\frac{\vert f\vert ^{p}}{ \theta^{p}}\,dV}\leq \biggl( \frac{N-p}{p} \biggr) ^{p}. $$

Since $\lim_{\epsilon \rightarrow 0^{+}}\int_{2\epsilon }^{ \pi }\theta^{-N}\sin ^{N-1}\theta \,d\theta \rightarrow +\infty $.

While for $1< p<2$, from (8), (9) and (10), one has

$$\begin{aligned}& \inf_{f\in C^{\infty }(S^{N})}\frac{C\int_{S^{N}}\vert f\vert ^{p}\,dV+ \int_{S^{N}}\vert \nabla_{S}f\vert ^{p}\,dV}{\int_{S^{N}}\frac{\vert f\vert ^{p}}{\theta ^{p}}\,dV} \\& \quad \leq \frac{C\int_{S^{N}}\vert f_{\epsilon }\vert ^{p}\,dV+\int_{S^{N}}\vert \nabla_{S_{\epsilon }}f\vert ^{p}\,dV}{\int_{S^{N}}\frac{\vert f_{\epsilon }\vert ^{p}}{ \theta^{p}}\,dV} \\& \quad \leq \frac{\vert S^{N-1}\vert \frac{\pi^{p}-\epsilon^{p}}{p}+ C_{4}+\vert S^{N-1}\vert ( \frac{N-p}{p} ) ^{p}\int_{2\epsilon }^{\pi }\theta^{-N}\sin ^{N-1}\theta \,d\theta }{\vert S^{N-1}\vert \int_{2\epsilon }^{\pi }\theta^{-N}\sin ^{N-1}\theta \,d\theta }, \end{aligned}$$

then passing to the limit as $\epsilon \rightarrow 0^{+}$, we have

12 $$ \inf_{f\in C^{\infty }(S^{N})}\frac{C\int_{S^{N}}\vert f\vert ^{p}\,dV+ \int_{S^{N}}\vert \nabla_{S}f\vert ^{p}\,dV}{\int_{S^{N}}\frac{\vert f\vert ^{p}}{\theta ^{p}}\,dV}\leq \biggl( \frac{N-p}{p} \biggr) ^{p}. $$

Therefore, from (2), (3), (11) and (12), we get that $( \frac{N-p}{p} ) ^{p}$ is the best constant of inequalities (2) and (3). Proof of Theorem 1 is finished.

## Conclusion

In this paper, we consider the Hardy type inequalities on the sphere. By the divergence theorem [14], we extend the results of Xiao [1] to a general case. We establish the $L^{p}$-Hardy inequalities on the sphere and obtain their best constants.
